# Endothelial Progenitor Cells Predict Cardiovascular Events after Atherothrombotic Stroke and Acute Myocardial Infarction. A PROCELL Substudy

**DOI:** 10.1371/journal.pone.0132415

**Published:** 2015-09-02

**Authors:** Elisa Cuadrado-Godia, Ander Regueiro, Julio Núñez, Maribel Díaz-Ricard, Susana Novella, Anna Oliveras, Miguel A. Valverde, Jaume Marrugat, Angel Ois, Eva Giralt-Steinhauer, Juan Sanchís, Ginès Escolar, Carlos Hermenegildo, Magda Heras, Jaume Roquer

**Affiliations:** 1 Department of Neurology, Neurovascular Research Group, IMIM-Hospital del Mar (Institut Hospital del Mar d'Investigacions Mèdiques), Universitat Autònoma de Barcelona/DCEXS-Universitat Pompeu Fabra, Barcelona, Spain; 2 Cardiology Department, Thorax Institute, Hospital Clínic, Universitat de Barcelona, Barcelona, Spain; 3 Cardiology Department, Hospital Clínico Universitario, Valencia.School of Medicine.Universitat de València, Valencia, Spain; 4 Hemotherapy-Hemostasis Department, Biomedical Diagnostics Center, Hospital Clínic, Universitat de Barcelona, Barcelona, Spain; 5 Valencia INCLIVA Biomedical Research Institute, Hospital Clínico, Valencia; Department of Physiology, Universitat de València, València, Spain; 6 Nephrology Department, Hospital del Mar. Universitat Autònoma de Barcelona, Barcelona, Spain; 7 Laboratory of Molecular Physiology and Channelopathies, Department of Experimental and Health Sciences, Universitat Pompeu Fabra, Barcelona, Spain; 8 Epidemiology and Cardiovascular Genetics Group. IMIM, Barcelona, Spain; University Francisco de Vitoria School of Medicine, SPAIN

## Abstract

**Introduction:**

The aim of this study was to determine prognostic factors for the risk of new vascular events during the first 6 months after acute myocardial infarction (AMI) or atherothrombotic stroke (AS). We were interested in the prognostic role of endothelial progenitor cells (EPC) and circulating endothelial cells (CEC)

**Methods:**

Between February 2009 and July 2012, 100 AMI and 50 AS patients were consecutively studied in three Spanish centres. Patients with previously documented coronary artery disease or ischemic strokes were excluded. Samples were collected within 24h of onset of symptoms. EPC and CEC were studied using flow cytometry and categorized by quartiles. Patients were followed for up to 6 months. NVE was defined as new acute coronary syndrome, transient ischemic attack (TIA), stroke, or any hospitalization or death from cardiovascular causes. The variables included in the analysis included: vascular risk factors, carotid intima-media thickness (IMT), atherosclerotic burden and basal EPC and CEC count. Multivariate survival analysis was performed using Cox regression analysis.

**Results:**

During follow-up, 19 patients (12.66%) had a new vascular event (5 strokes; 3 TIAs; 4 AMI; 6 hospitalizations; 1 death). Vascular events were associated with age (P = 0.039), carotid IMT≥0.9 (P = 0.044), and EPC count (P = 0.041) in the univariate analysis. Multivariate Cox regression analysis showed an independent association with EPC in the lowest quartile (HR: 10.33, 95%CI (1.22–87.34), P = 0.032] and IMT≥0.9 [HR: 4.12, 95%CI (1.21–13.95), P = 0.023].

**Conclusions:**

Basal EPC and IMT≥0.9 can predict future vascular events in patients with AMI and AS, but CEC count does not affect cardiovascular risk.

## Introduction

Endothelial progenitor cells (EPC) are mobilized from bone marrow and participate in adult neovascularization after both myocardial infarction [1] and ischemic stroke [2,3]. Circulating endothelial cells (CEC) on the other hand are mature cells that have detached from the intimal monolayer in response to endothelial injury [4]. Both types of cell are increased in patients with acute vascular disease [4–6]. However, the total count of these cells may be influenced by the presence of vascular risk factors traditionally associated with lower EPC and higher CEC counts [7–10] and the effects of medication to control these risk factors [11]. To date, no studies have analysed these two cell subtypes together in the same population.

In patients with stable coronary artery disease (CAD), EPC count has been associated with the risk of future cardiovascular events, mostly coronary revascularization [12,13]. However, the influence of these cells after acute myocardial infarction (AMI) is less well known. A previous study found the level of circulating EPCs to be predictive of poor neurological status and early stroke recurrence after ischemic stroke of various etiologies [14]. The role of CECs in both conditions has not been established.

The PROCELL study was a multicentre, prospective, population-based, case-control study paired by sex and age. The aim of the study was to compare the short- and long-term mobilization of EPCs and CECs following an AMI or an atherothrombotic stroke (AS), as this type of stroke shares the same etiology, large-vessel disease, and high cardiovascular risk of CAD [15,16]. Controls were recruited from a population-based cross-sectional study (REGICOR cohort study) [17], all free of selected cardiovascular risk factors (hypertension, dyslipidemia, diabetes), and matched by age and sex with cases. The main findings of this study have recently been published elsewhere [18]. In this substudy, we aimed to determine the effect of basal EPC and CEC on the risk of new vascular events (NVE) among high-risk vascular patients.

## Methods

The PROCELL study was a multicenter, prospective study performed in three tertiary hospital centres in Spain. The study was conducted according to the principles expressed in the Declaration of Helsinki. The study protocol was approved by the Institutional Ethics Committees (Hospital del Mar, Barcelona; Hospital Clínic, Barcelona; Hospital Clínico Universitario de Valencia) and all patients gave written informed consent before being included.

### Study population

Between February 2009 and July 2012, we included 100 consecutive patients with AMI—either ST elevation myocardial infarction (STEMI) or non-ST elevation MI (NSTEMI)—and 50 patients with acute ischemic stroke or transient ischemic attack (TIA) of atherothrombotic origin according to SSS-TOAST criteria [19] (presence of plaques in the symptomatic intracranial or extracranial artery with stenosis >50%, or stenosis <50% in a patient with more than one traditional vascular risk factor after excluding other embolic sources).

Inclusion criteria for AMI patients were ≤75 years of age, first AMI, and the presence of more than one traditional vascular risk factor. Exclusion criteria were documented CAD and previous treatment with statins. Inclusion criteria for AS were ≤75 years of age and initial stroke severity <20 on the National Institute of Health Stroke Scale (NIHSS). Exclusion criteria were previous documented stroke, previous disability >2 on the modified Rankin scale (mRS), and previous treatment with statins. We also excluded patients with kidney disease, active neoplasm, and chronic inflammatory or infectious diseases from both cohorts.

All patients underwent an initial arterial study that included a B-mode ultrasound of the carotid and vertebral arteries and transcranial Doppler ultrasound to assess intracranial circulation. A second arterial study confirmed the degree of stenosis, either by MRI angiogram (n = 36) or CT angiogram (n = 14). Degree of stenosis was categorized into four groups: 30% to 50%; 51% to 70%; 71% to 99%; and complete occlusion.

All patients received treatment according to current guidelines.

### Circulating endothelial cell and endothelial progenitor cell counts

Samples were collected in the first 24h after the initial AMI or AS. Blood was recovered in low molecular weight heparin tubes and processed in duplicate within 4h of extraction. An assessment of 2×10^6^ events per sample was considered sufficient for statistical analysis. EPC were defined as negative for CD45 and positive for CD34, KDR, and CD133 (CD45-CD34+KDR+CD133+). CEC were defined as negative for CD45 and positive for CD146 and CD31 (CD45-CD146+CD31+). We multiplied the EPC to CEC ratio obtained from flow cytometry analysis by the number of leukocytes/ml in the blood sample to obtain the absolute number of each cell type per 1 mL of whole blood.

### Variables analysed

We recorded traditional cardiovascular risk factors for all patients according to standard definitions: age, sex, hypertension, diabetes, hypercholesterolemia, body mass index (BMI), admission lipids and HbA1c levels, and previous medication. Other variables recorded were: common carotid artery (CCA) intima-media thickness (IMT), defined as the mean of three measurements in each carotid artery and measured using a semi-automated carotid ultrasound system (Sonosite MIcroMaxx IMT calc) according to international consensus [20]; and atherosclerotic burden (AB), determined on the basis of the number of affected vascular territories: coronary, cerebrovascular, and peripheral [21]. For the AB evaluation, all patients had carotid and transcranial Doppler ultrasounds (US). Cerebrovascular disease was diagnosed if >50% stenosis was found in the supra-aortic ultrasound. In stroke patients, CAD was evaluated with CT coronary angiography and diagnosed when stenosis exceeded 50%. Peripheral artery disease (PAD) was defined if the ankle-brachial index score was <0.9 or there was a previous history of intermittent claudication. Patients were thus classified into 3 groups according to the presence of disease in each territory. All data were available except IMT, which was not measurable in 17 patients (11.33%).

### Follow-up and vascular events

All patients had follow-up visits at 7, 30, 90, and 180 days. At each visit, any NVE was recorded. The study endpoints included new acute coronary syndrome, any TIA or stroke, and any hospitalization or death from cardiovascular causes. No patients were lost to follow-up.

### Statistical methods

Means, standard deviations and frequencies were computed to describe continuous, normally distributed, and categorical variables, respectively. Normality plots were constructed to check whether continuous variables followed normal distributions. EPCs and CECs were categorized into quartiles for further analysis. Student’s t-tests and ANOVA were performed to compare means between 2 or more groups, respectively, while chi-square tests were computed to compare frequencies between groups. Multivariate survival analysis was performed using Cox regression models to estimate the hazard ratio (HR) for NVE with 95% confidence intervals (CI). Multivariate models were adjusted for associated variables in the univariate analysis, considering a 2-sided P-value <0.05 as significant. Sensitivity analyses were performed to analyse potential confounding variables, due to the known association between age and a lower EPC count [22] and IMT with age and NVE [23]. Data analysis was performed using version 19 of the SPSS statistical program and was reviewed by a biostatistician.

## Results

### Study population

Of the 100 consecutive AMI patients, 87 presented with STEMI and 13 with non-STEMI. Coronary angiography was performed on all patients. In the STEMI group, primary percutaneous coronary intervention was the revascularization method used for 66 (76%) patients, and fibrinolysis for 15 (17%). Seven patients initially treated with fibrinolysis were subsequently treated with percutaneous coronary intervention. Of the 50 patients with acute cerebrovascular disease, 35 (70%) had a stroke diagnosis and 15 (30%) a TIA. The degree of stenosis in the symptomatic artery was 30% to 50% in 14 (34%) patients, 51% to 70% in 10 (20%), higher than 70% in 21 (42%) and complete occlusion in 2 (4%) patients. Two patients were treated with intravenous thrombolysis and 12 (24%) with delayed surgical or endovascular revascularization therapies.

Demographics and the clinical characteristics of the study population are shown in Table 1. Patients with AMI were younger (53.7 vs. 64.5 years; P <0.01) and showed a lesser prevalence of hypertension (27% vs. 68%; P< 0.01), diabetes mellitus (11% vs. 28%; P< 0.01), hypercholesterolemia (20% vs. 42%; P < 0.01) and a greater prevalence of cigarette smoking (74% vs. 56%; p = 0.03) than patients with strokes. Atherosclerotic burden was higher in patients with strokes than with AMI (36% with 3 territories vs 2%, P<0.01). Following discharge, AMI patients were more frequently treated with aspirin (99% vs. 78%; P< 0.001), clopidogrel (95% vs. 24%; P< 0.001), beta-blockers (85% vs. 6%; P< 0.001), and angiotensin-converting enzyme inhibitors (63% vs. 30%, P< 0.001) or angiotensin receptor blockers (26% vs. 14%, P = 0.09) than patients with strokes. The prescription of statins at discharge was similar among AMI and stroke patients (100% vs. 98%; P = 0.94). Anticoagulation therapy was very infrequent and was similar in both cohorts (8% of AMI vs 12% of strokes, P = 0.42).

**Table 1:** Bivariate analysis between AMI and stroke patients.

	AMI	STROKE	P value
	N = 100	N = 50	
**Age, mean (SD) y**	53.7(10.2)	64.5 (9.4)	<0.01
**Males, n (%)**	85 (85.0)	42 (84.0)	0.87
**Hypertension, n (%)**	27 (27.0)	34 (68.0)	<0.01
**Hypercholesterolemia, n (%)**	20 (20.0)	21 (42.9)	<0.01
**Diabetes Mellitus, n (%)**	11 (11.0)	14 (28.0)	<0.01
**Current smoking, n (%)**	74 (74)	28 (56)	0.03
**Previous IHD, n (%)**	0 (0)	2 (4)	0.04
**Previous stroke, n (%)**	0(0)	0 (0)	
**Previous PAD, n (%)**	2 (2)	2 (4)	0.474
**BMI**	26.6 (24.3–29.3)	28.1 (25.3–30.3)	0.117
**Total Cholesterol (mg/dL)**	197 (40)	199 (51)	0.80
**LDL Cholesterol (mg/dL)**	129 (33)	129 (39)	0.97
**Triglycerides (mg/dL)**	168 (135)	162 (88)	0.79
**HbA1c% (median, IQR)**	6.1 (1.7)	6.6 (1.6)	0.09
**AB, n (%)**			<0.01
**Three territories**	2 (2)	18 (36)	
**Two territories**	42 (42)	23 (46)	
**One territory**	56 (56)	9 (18)	
**IMT>0.9, n (%)**	31 (36.9)	20 (40.8)	0.65
**NVE, n (%)**	9 (9)	10 (20)	0.06

IHD = Ischemic heart disease

PAD = Peripheral arterial disease

BMI = Body mass index

AB = Atherosclerotic Burden

IMT = Intima media thickness

NVE = New vascular event.

### EPC and baseline clinical variables

Of the 150 patients, 14 (9.3%) had basal EPC samples that were unavailable or invalid and 18 (12%) had values of 0. The median and IQR for each EPC quartile were as follows: Q1, 0.0 (0.0–38.25); Q2, 97.5 (65.22–121.15); Q3, 214.65 (185.75–239.65), Q4, 486.60 (380.62–799.80). Higher basal EPC was associated with lower age (*p* for trend = 0.076), AMI (*p* = 0.019), and a lower proportion of DM (*p* = 0.077). Glycated haemoglobin was lower in patients with a low EPC, although all values were within the normal range. Although not statistically significant, patients with a higher AB had lower basal EPC. The univariate analysis is summarized in Table 2.

**Table 2:** Bivariate comparison between study variables and EPC quartiles.

	BasEPC Q1 N = 34	BasEPC Q2 N = 34	BasEPC Q3 N = 34	BasEPC Q4 N = 34	P value
**Age, mean (SD) y**	58.65 (10.50)	59.06 (12.29)	58.38 (10.28)	52.91 (11.08)	0.076
**Males, n (%)**	30 (88.2)	27 (79.4)	31 (91.2)	28 (82.4)	0.504
**AMI patients, n (%)**	20 (58.8)	19 (55.9)	22 (64.7)	30 (88.2)	0.019
**Stroke patients, n (%)**	14 (41.2)	15 (44.1)	12 (35.3)	4 (11.8)	
**Hypertension**	15 (44.1)	14 (41.2)	15 (44.1)	12 (35.3)	0.866
**Hypercholesterolemia, n (%)**	6 (17.6)	10 (29.4)	9 (26.5)	11 (32.4)	0.549
**Diabetes Mellitus, n (%)**	6 (17.6)	8 (23.5)	8 (23.5)	1 (2.9)	0.077
**Current smoker, n (%)**	25 (73.5)	20 (58.8)	23 (67.6)	25 (73.5)	0.517
**Alcohol overuse, n (%)**	8 (23.5)	6 (17.6)	7 (20.6)	2 (5.9)	0.227
**BMI, med (q25-q75)**	27.1(24.2–29.4)	26.6(24.4–29.8)	26.5(24.5–30.1)	27.4(25.8–29.4)	0.965
**HbA1c% med (q25-75)**	5.5 (5.0–5.9)	5.8 (5.6–6.7)	5.9 (5.3–7.5)	5.8 (5.4–6.0)	0.048
** AB, n (%)**					0.414
**Three territories**	7 (20.6)	4 (11.8)	6 (17.6)	1 (2.9)	
**Two territories**	13 (38.2)	17 (50.0)	13 (38.2)	17 (50.0)	
**One territory**	14 (41.2)	13 (38.2)	15 (44.1)	16 (47.1)	
**IMT>0.9, n (%)**	9 (29.0)	13 (41.9)	12 (38.7)	16 (55.2)	0.229
**NVE, n (%)**	9 (26.5)	3 (8.8)	2 (5.9)	3 (8.8)	0.020
**New stroke/TIA**	6 (17.6)	2 (5.9)	0 (0.0)	0 (0.0)	
**New ACS**	1 (2.9)	0 (0.0)	1 (2.9)	1 (2.9)	
**Other ACV event**	2 (5.9)	1 (2.9)	1 (2.9)	2 (5.9)	

BasEPC = Basal count of EPC

AMI = Acute myocardial infarction

BMI = Body mass index

AB = Atherosclerotic Burden

IMT = Intima-media thickness

NVE = New vascular event

ACS = Acute coronary syndrome

ACV = Acute cardiovascular event.

### CEC and baseline clinical variables

Of 150 patients included, 5 (3.3%) patients had no basal CEC data and 10 (6.7%) had a cell count of 0. The median and IQR of each CEC quartile were as follows: Q1, 21.50 (0.0–51.75); Q2, 113.97 (100.82–129.73); Q3, 208.30 (168.55–258.50); Q4, 570.50 (415.77–869.000). We found no association between cardiovascular risk factors or AB and basal CEC. Basal CEC count was higher in AMI patients (P = 0.014). A summary of the univariate analysis is found in Table 3.

**Table 3:** Bivariate comparison between study variables and CEC quartiles.

	BasCEC Q1 N = 34	BasCEC Q2 N = 34	BasCEC Q3 N = 33	BasCEC Q4 N = 34	P value
**Age, mean (SD)**	60.14 (10.26)	58.11(9.70)	54.22 (11.72)	56.94 (12.34)	0.145
**Males, n (%)**	29 (80.6)	31 (86.1)	32 (86.5)	31 (86.1)	0.877
**AMI patients, n (%)**	17 (47.2)	23 (63.9)	29 (78.4)	28 (77.8)	**0.014**
**Stroke patients, n (%)**	19 (52.8)	13 (36.1)	8 (21.6)	8 (22.2)	
**Hypertension, n (%)**	16 (44.4)	14 (38.9)	13 (35.1)	17 (47.2)	0.721
**Hypercholesterolemia, n (%)**	9 (25.0)	10 (27.8)	11 (29.7)	10 (27.8)	0.976
**Diabetes Mellitus, n (%)**	8 (22.2)	5 (13.9)	5 (13.5)	7 (19.4)	0.707
**Current smoker, n (%)**	20 (55.6)	29 (80.6)	27 (73.0)	23 (63.9)	0.117
**Alcohol overuse, n (%)**	6 (16.7)	10 (27.8)	4 (10.8)	4 (11.1)	0.176
**BMI med (q25-75)**	26.7(24.1–29.8)	26.8(24.3–29.4)	26.9(24.5–30.2)	27.5(26.1–29.6)	0.717
**HbA1c%, med (q25-75)**	5.8 (5.3–6.8)	5.7 (5.4–6.2)	5.8(5.4–6.4)	5.7 (5.4–6.2)	0.968
**AB, n (%)**					0.562
**Three territories**	6 (16.7)	3 (8.3)	5 (13.5)	5 (13.9)	
**Two territories**	16 (44.4)	21 (58.3)	13 (35.1)	15 (41.7)	
**One territory**	14 (38.9)	12 (33.3)	19 (51.4)	16 (44.4)	
**IMT>0.9, n (%)**	13 (43.3)	12 (35.3)	12 (37.5)	14 (42.4)	0.895
**NVE, n (%)**	7 (19.4)	6 (16.7)	3 (8.1)	2 (5.6)	0.219
**New stroke/TIA**	3 (8.3)	4 (11.1)	1 (2.7)	0 (0.0)	
**New ACS**	1 (2.8)	1 (2.8)	2 (5.4)	1 (2.8)	
**Other ACV event**	3 (8.3)	1 (2.8)	3 (8.1)	1 (2.8)	

BasCEC = Basal count of circulating endothelial cells

AMI = Acute myocardial infarction

BMI = Body mass index

AB = Atherosclerotic Burden

IMT = Intima media thickness

NVE = New vascular event

ACS = Acute coronary syndrome

ACV = Acute cardiovascular event.

### Incidence of cardiovascular events

A total of 19 patients (12.66%) had a NVE during follow-up (9/100 AMI patients and 10/50 stroke patients): 5 new strokes, 3 new TIA, 4 new AMI, 6 hospitalizations for other acute cardiovascular diseases, and 1 cardiovascular death. All new AMI patients had had a previous AMI and all the strokes/TIAs occurred in patients with initial stroke. Median time to NVE was 30 days (range 2–90). In univariate analysis, the incidence of NVE was associated with age (P for trend 0.039), IMT≥0.9 (P = 0.044) and basal EPC count (P = 0.041). The analysis is summarized in Table 4. Basal CEC count was not associated with NVE.

**Table 4:** Bivariate analysis between study variables and NVE.

	Vascular events yes N = 19	Vascular events No N = 131	P value
**Age, mean (SD)**	62.21 (9.74)	56.58 (11.17)	0.039
**Males, n (%)**	15 (78.9)	112 (85.5)	0.443
**Hypertension, n (%)**	7 (36.8)	54 (41.2)	0.758
**Hypercholesterolemia, n (%)**	6 (31.6)	35 (26.7)	0.653
**Diabetes Mellitus, n (%)**	4 (21.1)	21 (16)	0.587
**Current smoker, n (%)**	14 (73.7)	88 (67.2)	0.260
**BMI, med (q25-75)**	25.8 (23.7–19.4)	26.9 (24.8–29.7)	0.384
**Atherosclerotic Burden, n (%)**			0.205
**Three territories**	5 (26.3)	15 (11.5)	
**Two territories**	7 (36.8)	58 (44.3)	
**One territory**	7 (36.8)	58 (44.3)	
**IMT>0.9, n (%)**	9 (64.3)	42 (35.3)	0.044
**Basal EPC, n (%)**			0.041
**Q1**	9 (52.9)	25 (21.0)	
**Q2**	3 (17.6)	31 (26.1)	
**Q3**	2 (11.8)	32 (26.9)	
**Q4**	3 (17.6)	31 (26.1)	
**Basal CEC, n (%)**			0.219
**Q1**	7 (38.9)	29 (22.8)	
**Q2**	6 (33.3)	30 (23.6)	
**Q3**	3 (16.7)	34 (26.8)	
**Q4**	2 (11.1)	34 (26.8)	

BMI = Body mass index

IMT = Intima-media thickness

EPC = Endothelial progenitor cells

CEC = Circulating endothelial cells.

Regarding medication at discharge, the proportions of aspirin (79% vs 93%, P = 0.047), clopidogrel (52% vs.74%, p = 0.052) and beta-blockers given (42% vs. 61%, P = 0.094) was lower in patients who had had a NVE (Table A in S1 File).

To explore potential associations between each NVE and the study variables, we divided NVEs into two subgroups due to the low number of events in each category: the first group included new strokes/TIAs; and the second group, other vascular events (AMI/cardiovascular hospitalization or death). We performed a separate bivariate analysis between vascular risk factors and each subgroup outcome. Variables associated with new stroke/TIA compared with other vascular events were: HTA (75% vs. 9%, P = 0.014) and age (67.38 vs. 58.4, P = 0.026). Moreover, patients who had a new stroke/TIA had lower EPC counts than patients who suffered other vascular events (p = 0.027). (Table B in S1 File)

**Fig 1:**
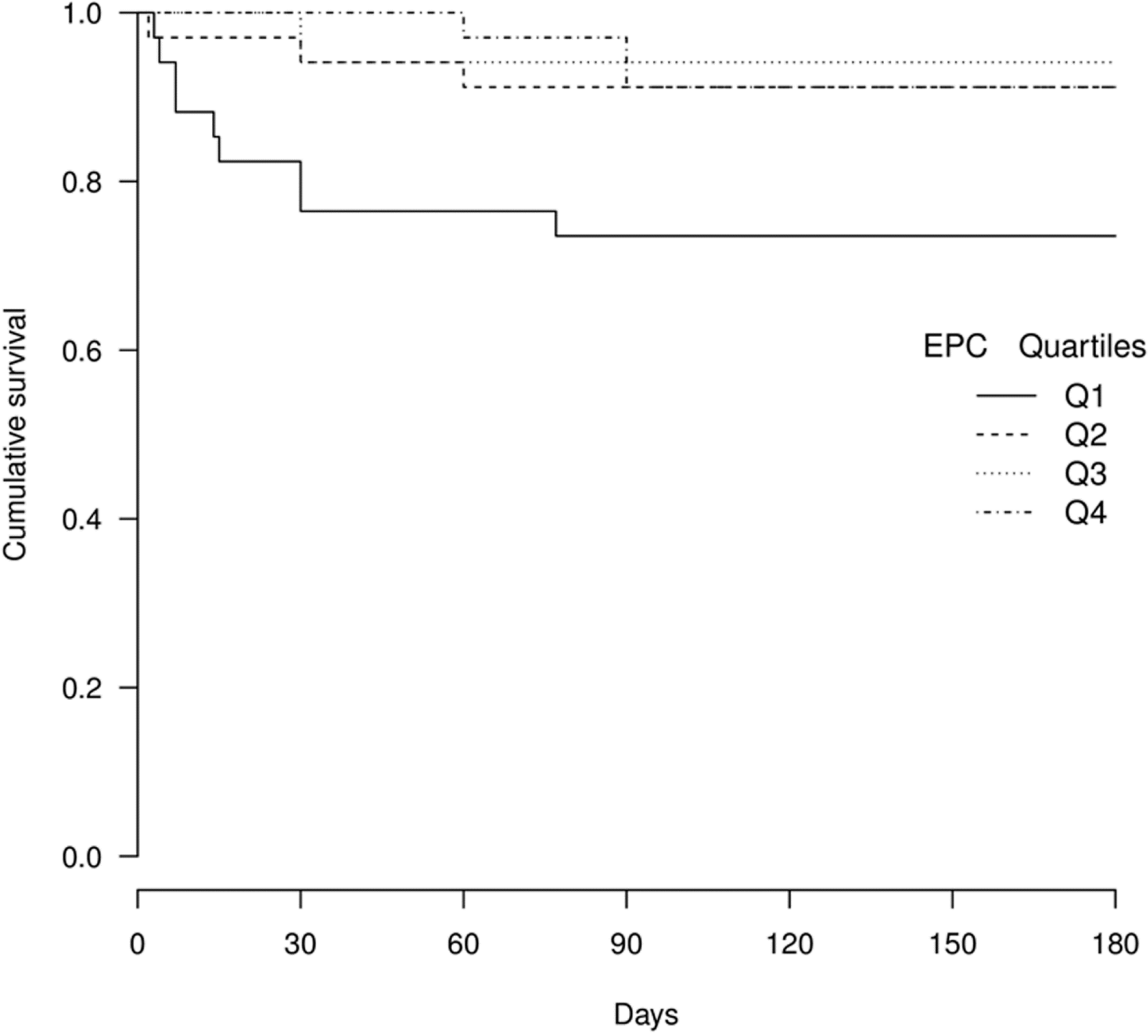
Kaplan-Meier survival curve according to EPC quartiles.

Multivariate Cox regression analysis showed an independent association with basal EPC. The risk is highest in the lowest quartile (Table 5 and Fig 1). Compared with Q4, adjusted HRs were as follows: Q1 [HR: 10.33, 95% CI (1.22–87.34), P = 0.032]; Q2 [HR: 1.43, 95%CI (0.17–17.48) P = 0.778]; Q3 [HR: 0.67, 95% CI (0.03–11.48), P = 0.789]. IMT≥0.9 was also an independent predictor of NVE [HR: 4.12, 95% CI (1.21–13.95), P = 0.023]. In the model without IMT but adjusted for age (Table C in S1 File), sensitivity analyses showed an estimated HR of 2.96 (p = 0.105) for EPC Q4 vs Q1, whereas in the model without age but adjusted for IMT (Table C in S1 File), the HR was 15.77 (p = 0.010). These results clearly indicate that age was not a confounding variable when IMT was already present in the model, since the HR remained significant and varied from 10.33 to 15.77 (Table 4). On the other hand, IMT was a strong reverse confounding variable, even in the presence of age, since the HR varied from 10.33 to 2.96 and ceased to be significant when IMT was removed from the model.

**Table 5:** Cox regression analysis for the risk of NVE.

	HR 95% CI	P
**Basal EPC quartiles**		0.003
**Basal EPC Q1**	10.33 (1.22–87.34)	0.032
**Basal EPC Q2**	1.43 (0.17–17.48)	0.778
**Basal EPC Q3**	0.67 (0.03–11.48)	0.786
**IMT > 0.9**	4.12 (1.21–13.95)	0.023
**Age**	1.05 (0.98–1.12)	0.118

EPC = Endothelial progenitor cells

IMT = Intima-media thickness.

Other variables associated in bivariate analysis, such as HbA1c or medication at discharge, were not associated in the Cox regression analysis. (Tables D-E in S1 File)

## Discussion

Our study is the first to show that basal EPC, but not basal CEC count, is associated with 6-month NVE in patients with AMI and AS. Cerebrovascular diseases and CAD are the highest causes of mortality worldwide. However, whereas CAD is usually attributable to large-vessel atherosclerosis, stroke has a far more heterogeneous pathophysiology, including emboli originating from the heart, cerebral small-vessel disease (lacunar infarcts), and an abundant variety of other less frequent causes.

Atherothrombotic strokes—the most prevalent stroke subtype [24]—are those caused by large-vessel atherosclerosis in the major blood vessels supplying the brain, such as the carotid, vertebral, and basilar arteries or the vessels forming the circle of Willis. This stroke subtype and AMI not only share a common pathophysiology (large-vessel atherosclerosis) but also a high (20%) 10-year risk of future cardiovascular events [15,16].

Although very promising results have been found in trials using stem cell therapy in vascular diseases [25,26], the role of circulating EPC and CEC is not completely understood. The PROCELL study aimed to study the role of these cells in patients with AMI and acute AS.

We found that patients with a very low EPC count have a higher risk of a NVE during the first 6 months. These results come as no surprise in AMI patients since previous studies have found similar results. In a large study including 519 patients undergoing coronary angiography [27], decreasing levels of baseline EPC were associated with death from cardiovascular causes, first major cardiovascular events, revascularization and hospitalization after 12 months of follow-up, although EPCs were not associated with AMI or stroke. That study included patients with acute, subacute and chronic myocardial infarction and the incidence of AMI or stroke during follow-up was less than in our study (6.7% and 3.4% respectively). There was also no information about stroke subtype (ischemic or haemorrhagic). Another study including 44 patients with stable CAD, 33 patients with acute coronary syndrome and 43 control subjects also found an independent association with EPC and later cardiovascular events during a median follow-up of 10 months [13]. Eleven patients (14.5%) had a cardiovascular event, but most of these were revascularizations

In addition, a lower EPC count has been associated with NVEs in healthy subjects [28] and patients with kidney disease [29] or metabolic syndrome [30]. However, in most of these studies, a new stroke was not considered an outcome.

There are numerous differences between these studies and ours: We included only patients with acute diseases (AMI and stroke) because these patients are at a high risk of subsequent cardiovascular events. [31] For this reason, we studied patients admitted within the first 24h after the initial event and the follow-up was shorter. It is worthy of note that, in our study, 6 of 19 patients (31.57%) had the NVE in the first week of follow-up, pointing out the importance of studying acute patients. Furthermore, we studied ischemic strokes that had the same etiology, large artery atherosclerosis, whereas in the previous studies, the stroke etiologies were mixed; this is a crucial matter, especially when studying pathophysiology. We did not include programmed revascularization as an outcome because we focused only on the risk of unexpected acute cardiovascular events. We included ischemic stroke as an outcome, whereas other studies did not. Finally, our study is the first to consider EPC and CEC counts in the same cohort.

In addition, to the best of our knowledge, the association between NVE and basal EPC count has not previously been described in stroke patients. Only one study [14] found an association between lower basal EPC and a combination of NVE and poor neurological status at 90 days, with only 3 out of 138 patients having a stroke recurrence.

Several experimental studies have proved that circulating EPC is capable of mobilizing to injured arteries and repairing the endothelium [32]. In our study, we also observed the potential protective effect of these cells as a marker of a high risk of vascular events even in patients receiving the best medical treatment available.

Although the role of EPC has been broadly studied, the influence of CEC on the risk of NVE has received little attention. The association between CEC and NVE in patients with ischemic stroke has not been previously reported, and we found only one previous study of patients with acute coronary syndrome in which 48h CEC was the only independent predictor of a major cardiovascular event at one year [33]. We were unable to replicate these findings; this was probably due to methodological differences between the studies, most importantly that the CEC characterization was different (CD 146+CD31- in the study cited and CD146+CD31+ in our study) and also that strokes were not included as a vascular outcome in the earlier study.

Apart from the cell analysis, we aimed to identify other clinical variables with a predictive role in vascular risk. We found that carotid IMT≥0.9 was independently associated with the risk of NVE. Increased carotid IMT has been considered an early marker of atherosclerosis and a proven risk factor for future vascular events in healthy populations, as well as in patients with cardiovascular disease, independently of other vascular risk factors [23,34]. In our study, IMT≥0.9 was more informative than other clinical variables such as age, vascular risk factors, or atherosclerotic burden. This is important because, although various meta-analyses found that IMT measurement adds little predictive value in the general population [35,36], it can help identify subgroups with a worse prognosis in patients at high vascular risk, such as AMI or AS patients.

Our study has several strengths. We have uniformly studied patients with different acute manifestations of a common disease, large-vessel atherosclerosis. All patients underwent exhaustive vascular study and were treated under current international guidelines. All samples were collected within the first 24h of the ischemic event in order to study early recurrences. Finally, this was the first study to analyse both cell subtypes at the same time. The study also has a few limitations, the most important being the small sample size. However it should be pointed out that the PROCELL study was a prospective, multicenter case-control study that included a highly selected population. Patients were studied during the acute phase and the follow-up was very thorough [18]. In addition, the results of the study are in agreement with previous studies with similar methodologies and similar sample sizes. [13,14,33] We consider therefore that although the small sample size prevented a more detailed analysis of patient subtypes and vascular events, it does not invalidate the association between EPC and NVE. Secondly, although the information on the patients recruited was extensive, we did not include other comorbidities that may have influenced the association between EPC and the risk of NVE, such as obstructive sleep apnea, which is implicated in the development of hypertension, endothelial dysfunction and higher intima-media thickness, all elements known to lead to atherosclerosis. [37] Lastly, although the medication prescribed and patient compliance was verified at every visit, due to the observational nature of the study and the study size, the influence of drugs on the risk of vascular events could not be ascertained.

## Conclusions

In patients with acute manifestations of large-vessel atherosclerosis, such as AMI or AS, a low EPC count is a strong predictor of future vascular events, whereas CEC count has no predictive role. More research on this field is needed to confirm the protective role of EPC in patients with vascular disease.

## Supporting Information

S1 File Table A. Bivariate analysis of medication at discharge and the risk of new vascular events. Table B. Bivariate analysis of study variables and subtypes of NVE. Table C. Model A. Sensitivity analysis: Cox regression model after removal of IMT. Model B. Cox regression model after removal of age. Table D. Cox regression analysis including HbA1c. Table E. Cox regression analysis including aspirin.
(DOCX)
