# Supplementary material for: Endothelial Progenitor Cells Predict Cardiovascular Events after Atherothrombotic Stroke and Acute Myocardial Infarction. A PROCELL Substudy
Source: PLoS One. 2015 Sep 2;10(9):e0132415. doi: 10.1371/journal.pone.0132415 (PMC4557832; doi:10.1371/journal.pone.0132415)
Supplement: S1 File — Bivariate analysis of medication at discharge and the risk of new vascular events. Table B. Bivariate analysis of study variables and subtypes of NVE. Table C. Model A. Sensitivity analysis: Cox regression model after removal of IMT. Model B. Cox regression model after removal of age. Table D. Cox regression analysis including HbA1c. Table E. Cox regression analysis including aspirin. (DOCX) [file pone.0132415.s001.docx]

**Table A. Bivariate analysis of medication at discharge and the risk of new vascular events**

|  | **Vascular events yes**  **N=19** | **Vascular events No**  **N=131** | **P value** |
| --- | --- | --- | --- |
| **Aspirin** | 15 (78.9) | 123 (93.3) | 0.047 |
| **Clopidogrel** | 10 (52.6) | 97 (74) | 0.052 |
| **Oral anticoagulants** | 0 (0) | 10(7.6) | 0.247 |
| **ACE inhibitors** | 8 (42.1) | 70 (53.4) | 0.249 |
| **Beta-blockers** | 8 (42.1) | 80 (61.1) | 0.094 |
| **ARB** | 3 (15.58) | 30 (22.9) | 0.358 |
| **Statins** | 18 (94.7) | 130 (99.2) | 0.238 |

**Table B. Bivariate analysis of study variables and subtypes of NVE**

|  | | **No Events**  **N=131** | **Cerebral event**  **N=8** | **Other CV event**  **N=11** | **P value** |
| --- | --- | --- | --- | --- | --- |
| **Age (years)** | 56.5 (11.1) | 67.38 (8.2) | 58.4 (9.27) | 0.026 |  |
| **Males** | 112 (85.5) | 7 (87.5) | 8 (72.7) | 0.515 |  |
| **Hypertension** | 54 (41.2) | 6 (75.0) | 1 (9.1) | 0.014 |  |
| **Hypercholesterolemia** | 35 (26.7) | 3 (37.5) | 3 (27.3) | 0.802 |  |
| **Diabetes Mellitus** | 21 (16.0) | 3 (37.5) | 1 (9.1) | 0.224 |  |
| **Current smoker** | 88 (67.2) | 5 (62.5) | 9 (81.8) | 0.572 |  |
| **Atherosclerotic Burden** |  |  |  | 0.296 |  |
| **Three territories** | 15 (11.5) | 3 (37.5) | 2 (18.2) |  |  |
| **Two territories** | 58 (44.3) | 2 (25.0) | 5 (45.5) |  |  |
| **One territory** | 58 (44.3) | 3 (37.5) | 4(36.4) |  |  |
| **IMT>0.9** | 42 (35.3) | 5 (62.5) | 4 (66.7) | 0.107 |  |
| **BMI** | 26.9 (24.8-29.7) | 29.0 (24.5-32.8) | 25.7 (23.5-27.7) | 0.137 |  |
| **Basal EPCs** |  |  |  | 0.027 |  |
| **Q1** | 25 (31.0) | 6 (75.0) | 3 (33.3) |  |  |
| **Q2** | 31 (26.1) | 2 (25.0) | 1 (11.1) |  |  |
| **Q3** | 32 (26.9) | 0 (0) | 2 (22.2) |  |  |
| **Q4** | 31 (26.1) | 0 (0) | 3 (33.3) |  |  |

**Table C. Sensitivity analysis: Cox regression model after stepwise removal of associated variables. Model A: Cox regression model after removal of IMT. Model B: Cox regression model after removal of age.**

**MODEL A**

|  | **HR 95% CI** | **P value** |
| --- | --- | --- |
| **Basal EPC quartiles** |  | 0.038 |
| **Basal EPC Q1** | 2.96 (0.80-10.98) | 0.105 |
| **Basal EPC Q2** | 0.72 (0.14-3.71) | 0.693 |
| **Basal EPC Q3** | 0.51 ( 0.08 - 3.09) | 0.462 |
| **Age** | 1.06 (1.00 - 1.11) | 0.038 |

**MODEL B**

|  | **HR 95% CI** | **P value** |
| --- | --- | --- |
| **Basal EPC quartiles** |  | 0.002 |
| **Basal EPC Q1** | 15.77 (1.95-126.98) | 0.010 |
| **Basal EPC Q2** | 2.41 (0.21-26.67) | 0.473 |
| **Basal EPC Q3** | 1.20 (0.75-17.19) | 0.896 |
| **IMT > 0.9** | 5.20 (1.57- 17.19) | 0.007 |

**Table D. Cox regression analysis including HbA1c**

|  | **HR 95% CI** | **P value** |
| --- | --- | --- |
| **Basal EPC quartiles** |  | 0.003 |
| **Basal EPC Q1** | 10.33 (1.22-87.34) | 0.032 |
| **Basal EPC Q2** | 1.43 (0.17-17.48) | 0.778 |
| **Basal EPC Q3** | 0.67 (0.03-11.48) | 0.786 |
| **IMT > 0.9** | 4.12 (1.21-13.95) | 0.023 |
| **Age** | 1.05 (0.98-1.12) | 0.118 |
| **HbA1c** | 1.03 (0.73-1.40) | 0.843 |

**Table E. Cox regression analysis including Aspirin**

|  | **HR 95% CI** | **P value** |
| --- | --- | --- |
| **Basal EPC quartiles** |  | 0.004 |
| **Basal EPC Q1** | 9.88 (1.16-83.81) | 0.036 |
| **Basal EPC Q2** | 1.32 (0.10-16.66) | 0.826 |
| **Basal EPC Q3** | 0.71 (0.04-12.39) | 0.815 |
| **Age** | 1.05 (0.98-1.12) | 0.154 |
| **IMT > 0.9** | 4.15 (1.23-13.99) | 0.022 |
| **Aspirin** | 0.65 (0.17-2.55) | 0.546 |
